# TransLiteUNet: A Lightweight CNN–Transformer Hybrid for Efficient 3D Brain Tumor Segmentation with Sub-0.5 M Parameters

**DOI:** 10.3390/jimaging12070290

**Published:** 2026-06-30

**Authors:** Lixin Zhou, Yuanyuan Yang, Yunfeng Yang

**Affiliations:** 1 Laboratory for Medical Imaging Informatics, Shanghai Institute of Technical Physics, Chinese Academy of Sciences, Shanghai 200083, China; zhoulixin@mail.sitp.ac.cn (L.Z.); yangyuanyuan@mail.sitp.ac.cn (Y.Y.); 2 Laboratory for Medical Imaging Informatics, University of Chinese Academy of Sciences, Beijing 100049, China

**Keywords:** Transformer, encoder–decoder, lightweight model, medical volumetric segmentation, TransLiteUNet

## Abstract

Transformer, with its unique self-attention mechanism, naturally excels in modeling global features. Convolutional Neural Networks (CNNs), on the other hand, leverage strong spatial inductive biases to effectively capture local features with fewer parameters. In 3D brain tumor segmentation, both local and global features are critical. Moreover, balancing high accuracy with computational cost in 3D segmentation models remains a key challenge. To address this, we propose TransLiteUNet, a lightweight 3D solution that combines CNN and Transformer architectures for accurate brain tumor segmentation without pretraining. To enhance parameter efficiency, we introduce a 3D axial depthwise separable convolution residual structure (3DRes-ADS block) and a lightweight LiteViT module, which improves global feature modeling at a lower computational cost. Specifically, TransLiteUNet (0.43 M parameters, 14.98 GFLOPs) and its simplified version, TransLiteUNet-S (0.31 M parameters, 7.68 G FLOPs), offer significantly lower model complexity compared to current state-of-the-art models. Tested on two publicly available datasets, our models outperform leading models under identical conditions. The parameter and computational costs are reduced by orders of magnitude, with optimized inference and training costs.

## 1. Introduction

Gliomas are the most common and highly invasive malignant brain tumors, posing significant challenges to diagnosis, treatment, and prognosis [1]. Multimodal MRI-based automatic segmentation techniques enable precise delineation of anatomical structures and lesion regions, which is crucial for diagnosis, treatment planning, and disease monitoring [2].

The advent of Convolutional Neural Networks (CNNs), particularly Fully Convolutional Networks (FCNs) [3], has greatly advanced the field of medical image segmentation. The subsequent U-Net model [4], with its effective encoder–decoder architecture and skip connections, significantly improved segmentation accuracy and detail, becoming the preferred choice among researchers. Despite CNN’s substantial advantages, its inherent locality due to convolution operations limits long-range modeling, a drawback that is amplified when there are significant differences in texture, size, or shape between segmented objects. Recognizing this limitation, researchers have increasingly turned their attention to Transformer-based models [5].

The Transformer architecture [6], initially designed for natural language processing, has shown significant success in various computer vision tasks due to its efficient global self-attention mechanism, which excels at capturing global information [7,8]. However, pure Transformer models typically require large parameter scales and extensive pretraining on large datasets to converge, which limits their application in medical image segmentation, as labeled large-scale medical image data are often scarce. Transformer-based models generally require extensive parameter training to achieve optimal performance, likely due to the lack of image-specific inductive biases, a characteristic inherent to CNNs [9]. On the other hand, compared to 2D segmentation networks, 3D segmentation networks offer improved accuracy and detail in tumor segmentation tasks [10], yet they also entail larger input data dimensions and higher computational complexity, resulting in models with substantial parameters and computational demands [10,11,12,13,14].

To address this, researchers have proposed hybrid architectures that combine the strengths of CNNs and Transformers, aiming to leverage CNN’s spatial inductive bias and Transformer’s global context modeling capability [13,15,16,17]. While these models mitigate CNN’s limitations in global modeling, achieving a balance between model complexity and performance remains a critical challenge. In theory, high-parameter models offer better performance but often come with higher computational costs, which are disadvantageous for practical deployment [18,19,20]. Recent work, such as FNIRNet [21], further demonstrates that lightweight deep learning frameworks with compact architectures can achieve high classification performance in brain-related clinical tasks while supporting practical deployment in resource-constrained settings, reinforcing the necessity of developing parameter-efficient models for neurological applications.

Research Motivation: To address the challenge of balancing model complexity and performance, we propose a lightweight and efficient 3D brain tumor segmentation model, TransLiteUNet. This model introduces the novel 3D Res-ADS module, based on 3D axial depthwise separable convolutions, significantly reducing computational load while maintaining the ability to capture detailed image features. Inspired by the ConvNeXt model [22], we utilized a 7 × 7 × 7 kernel to achieve a larger global receptive field. Additionally, we designed a lightweight Transformer architecture, the LiteViT module, to enhance the model’s global context modeling capability.

Notably, TransLiteUNet and TransLiteUNet-S achieve accurate medical image segmentation without any pretraining, with only 0.43 M parameters and 14.98 GFLOPs in terms of computational complexity. Compared to state-of-the-art 3D convolutional models like 3D UX-Net [23] and the CNN–Transformer hybrid CKD-TransBTS [14], these models significantly reduce computational complexity while achieving superior performance in 3D brain tumor segmentation. Through 5-fold cross-validation on the BraTS2020 and BraTS2019 datasets, the proposed models consistently outperform existing advanced models in terms of average Dice scores on the test set. Extensive model comparisons confirm that TransLiteUNet and TransLiteUNet-S represent efficient solutions for 3D brain tumor segmentation and establish a new benchmark for lightweight 3D multimodal brain tumor segmentation models. The contributions of this paper are summarized as follows:

1. 3D Res-ADS Module for Lightweight 3D Brain Tumor Segmentation: Inspired by U-Lite and ConvNeXt, we propose the 3D Res-ADS module, which leverages depthwise separable convolutions and residual learning. This module employs novel 3D axial convolutions and a 7 × 7 × 7 large convolution kernel design to enhance feature extraction, expand the receptive field, and reduce computational complexity.
2. LiteViT Module for Global Feature Modeling: To improve global feature modeling, we introduce the LiteViT module, which extracts global information from images. Building upon the original MobileViT module, we replace the 3 × 3 × 3 local convolution blocks with 7 × 7 × 7 3D Res-ADS layers. This allows for efficient global representation learning with minimal parameters, complemented by learnable positional encodings in the Transformer component for enhanced global modeling.
3. TransLiteUNet and Its Simplified Version, TransLiteUNet-S: We propose TransLiteUNet and its simplified version, TransLiteUNet-S, which achieve state-of-the-art segmentation results across multiple public datasets. This ultra-lightweight, structurally simple hybrid architecture based on CNN and Transformer minimizes unnecessary innovations and optimizes existing modules for optimal performance.

## 2. Related Work

U-Net [4] is a pioneering work in medical image segmentation, consisting of a symmetric encoder–decoder network. The encoder progressively reduces spatial dimensions and extracts features, while the decoder restores the spatial resolution and generates the segmentation result. The U-Net model utilizes skip connections to combine features from the encoder with the decoder, effectively leveraging both low-level and high-level features to enhance segmentation accuracy. Since its introduction, numerous subsequent works have been developed based on U-Net, such as U-Net++ [24], U-Net3+ [25], 3D U-Net [10], and V-Net [11]. Additionally, Schlemper further incorporated a gated attention mechanism into the U-Net, proposing the Attention Gated Network, which dynamically adjusts feature representations through attention mechanisms to focus on more important image regions, thus improving segmentation accuracy and smoothness [12].

The Vision Transformer (ViT) [7] was the first model to effectively adapt the widely used Transformer architecture from natural language processing to image recognition, achieving revolutionary success. In ViT, an image is first divided into patches, each of which is flattened and augmented with positional encoding before being fed into the Transformer module. The self-attention mechanism captures the relationships between different patches, enabling the model to learn rich visual features. The subsequent Swin Transformer [8] further reduces computational complexity by limiting self-attention to non-overlapping local windows of size 7 × 7.

ConvNeXt [22], a recently proposed efficient pure convolutional network model inspired by ViT, redefines the standard ResNet [26] architecture, identifying several key components that impact model performance (Macro Design, Inverted Bottleneck, Large Kernel, and Micro Design). ConvNeXt competes with Transformer models in terms of accuracy and scalability, achieving an impressive 87.8% ImageNet Top-1 classification accuracy while maintaining a simple architecture.

The concept of depthwise separable convolution was first introduced in 2014, with significant improvements made by Chollet [27], who proposed the Xception architecture, fully based on depthwise separable convolutions. This convolution operation decomposes the traditional convolution process into two steps: depthwise convolution and pointwise convolution. This approach significantly reduces both computational cost and parameter count. Many subsequent lightweight models have been designed based on depthwise separable convolutions.

MobileViT [19], proposed by Apple, is a lightweight CNN–Transformer hybrid architecture that captures long-range dependencies and global information while maintaining low computational complexity. MobileViT has been validated on multiple computer vision tasks, demonstrating superior performance on mobile devices, particularly in applications requiring efficient image analysis, such as medical image segmentation and object detection. Subsequent research has led to improved versions, such as MobileViT v2 [28] and MobileViT v3 [29].

In recent years, MLP-based network architectures have gained prominence, particularly in image processing [30,31]. One of the earliest MLP models, MLP-Mixer [30], employs a network architecture composed entirely of MLPs for image classification. The subsequent Vision Permutator [32] advocates for encoding features along the height and width dimensions via linear projections, enabling the model to capture long-range dependencies while avoiding the attention mechanism of Transformers. Inspired by Vision Permutator and ConvNeXt, Dinh et al. [20] proposed the U-Lite, a lightweight model for 2D medical image segmentation. With only 878 K parameters, U-Lite outperforms U-Net and its variants (such as Unet++ [24], ConvUNeXt [33], and UneXt [34]). This impressive performance is largely attributed to its proposed 2D axial depthwise separable convolution module, which not only expands the receptive field but also enhances feature extraction capabilities, making it especially suited for detail extraction and precise segmentation in medical images. The design of the 3D Res-ADS module in this study is inspired by the 2D axial depthwise separable convolution.

## 3. Proposed Model

### 3.1. The Overall Architecture of TransLiteUNet

**Figure 1:**
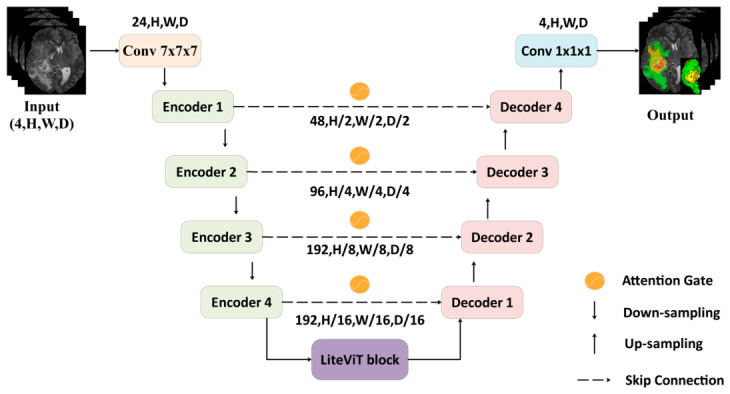
Schematic of the overall architecture of the TransLiteUNet model. The color-coded regions in the upper right indicate the target segmentation areas: yellow denotes the enhancing tumor, red denotes the necrotic and non-enhancing tumor, and green denotes the peritumoral edema.

Figure 1 illustrates the overall architecture of TransLiteUNet, which follows a symmetric encoder–decoder structure based on the U-Net framework. A multi-modal MRI sequence input $X\in R^{C\times H\times W\times D}$ is used, where the depth dimension is *D* (representing different image slices), the number of channels is *C* (representing different image sequence modalities), and the resolution is H×W. We first use a 3D CNN (3D Res-ADS module and MaxPool3d) downsampling module to generate feature maps containing spatial features of the MRI images. Then, the LiteViT module reshapes each volume into vectors (tokens) and feeds them into a Transformer architecture for global feature modeling. The output of the LiteViT module matches the shape of the output feature map. Subsequently, the 3D decoder iteratively stacks upsampling layers and 3D CNN layers (dual 3D Res-ADS modules) while employing an attention gate mechanism for skip connections to capture low-level features. Finally, the full-resolution segmentation result is obtained. The design of TransLiteUNet is simple, and its lightweight and high-performance advantages are mainly attributed to the 3D Res-ADS and LiteViT modules.

### 3.2. The 3D Res-ADS Module

**Figure 2:**
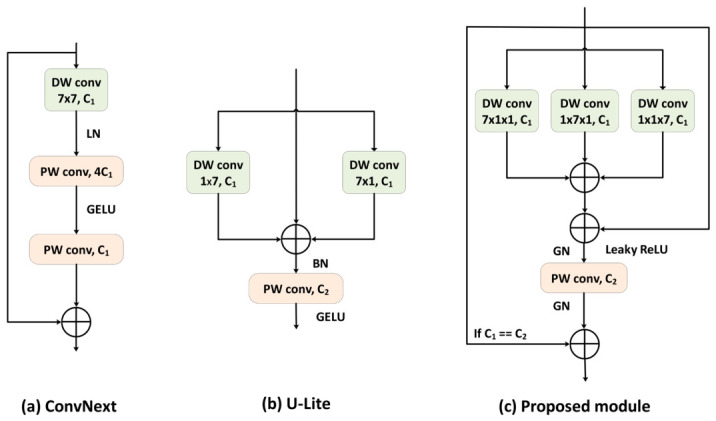
Schematic of convolution module strategies in different networks: (**a**) ConvNeXt model; (**b**) U-Lite model; (**c**) lightweight module proposed in this study.

To effectively reduce the parameter size and computational complexity of CNN models, various lightweight strategies have been proposed. MobileNet [35] reduces the computational cost and parameters of convolutional layers by using depthwise separable convolutions. ShuffleNet [36] employs channel shuffle and pointwise group convolution techniques to enhance the flow of image features across channels and reduce the computational complexity of convolution operations, improving model efficiency. EfficientNet [37] optimizes depth, width, and resolution through a compound scaling strategy, significantly balancing efficiency and accuracy. ConvNeXt [22] reaffirms the advantages of the 7 × 7 large convolution kernel, with its structural design shown in Figure 2a. U-Lite [20] brings the parameter size of the 2D U-Net segmentation model below 1 M for the first time, with experimental results further validating the power of axial depthwise separable convolutions, as illustrated in Figure 2b.

Although significant breakthroughs have been made in lightweight and efficient models for 2D image tasks, such models are notably lacking for 3D image segmentation tasks. Existing 3D medical image segmentation models often prioritize segmentation performance, leading to models with high complexity [13,14,16,38]. To address this, we propose a simple yet efficient 3D convolutional architecture that achieves optimal 3D feature fusion with minimal computational cost. Inspired by ConvNeXt and U-Lite networks, we introduce a simple 3D convolution module (3D Res-ADS module), as shown in Figure 2c, with the following mathematical expression:

(1) $$x\prime =x+DW_{n\times 1\times 1}(x)+DW_{1\times n\times 1}(x)+DW_{1\times 1\times n}(x)$$

(2) y=x+GN(PWC1→C2LeakyReLuGNx′           if C1==C2GN(PWC1→C2LeakyReLuGNx′    else

In this equation, *x* represents the input features, and *y* represents the output features; *DW*, *PW*, and *GN* correspond to depthwise convolution, pointwise convolution, and group normalization (GN), respectively. n×1×1, 1×n×1, and 1×1×n denote the kernel sizes, where n = 7; *C*_1_ and *C*_2_ denotes the input and output channel numbers. As shown in Equation (2), a residual connection is added when $C_{1}==C_{2}$ (i.e., the input and output channel numbers are equal); otherwise, the residual connection is omitted, and only the transformed features are output. Notably, when the input and output channels are identical, the 3D Res-ADS block functions as a plug-and-play module, preserving the shape of the input feature map while performing feature extraction and fusion. As Dinh et al. [20] pointed out, axial convolutions offer a larger receptive field with fewer computational parameters compared to standard convolution operators.

### 3.3. Encoder Module

**Figure 3:**
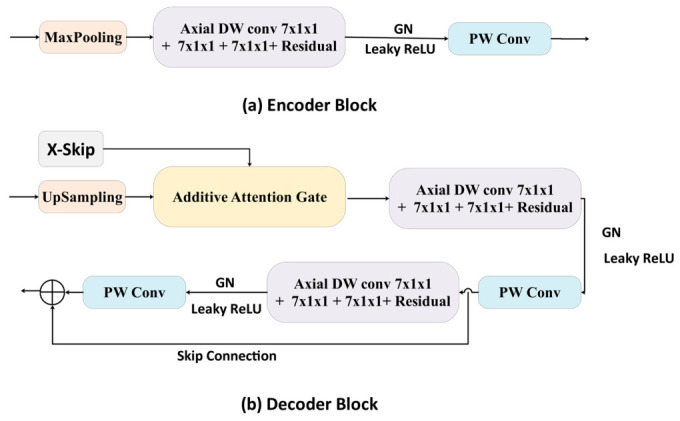
Architecture of the encoder and decoder in TransLiteUNet: (**a**) encoder of TransLiteUNet; (**b**) decoder of TransLiteUNet.

Due to the quadratic relationship between the computational complexity of Transformer models and the number of tokens, directly flattening images for Transformer input incurs a significant computational cost, especially for 3D images. To better leverage the strengths of both CNN and Transformer architectures while effectively controlling computational cost, the encoder in this work combines the 3D Res-ADS module and MaxPooling layer to progressively encode 3D images into low-resolution high-level feature representations $F\in R^{K\times \frac{H}{16}\times \frac{W}{16}\times \frac{D}{16}}$ (*K* = 192). This approach efficiently captures local 3D context information, yielding compact volumetric feature maps. The specific structure of the encoder is shown in Figure 3a, where a 3D Res-ADS module without residual connections follows MaxPooling. This structure incurs very low computational cost. A detailed breakdown of the parameter calculation for each layer and module is provided in Table S1, where the entire encoder module (comprising initial convolution through LiteViT block) accounts for only 0.248 M parameters (approximately 0.071 M when considering only the downsampling and encoding operations). The parameter calculation for each module follows standard conventions: for a 3D convolutional layer with kernel size K, input channels $C_{in}$, and output channels $C_{out}$, the number of parameters is $K^{3}C_{in}C_{out}+C_{out}$ (including bias). For the depthwise separable convolutions used in the 3D Res-ADS module, the parameters are calculated as $K^{3}C_{in}$ for the depthwise step and $C_{in}C_{out}+C_{out}$ for the pointwise step. For the Transformer component in the LiteViT module, the parameters come from the multi-head self-attention projections and the feed-forward network, with detailed per-layer calculations reflected in Table S1. The encoded features are then input into the LiteViT module for further learning of long-range dependencies in the global receptive field.

### 3.4. LiteViT Module

With the successful application of ViT models in vision tasks, researchers have continuously refined this novel paradigm to achieve better performance. The Swin Transformer is one such improvement, which reduces the computational complexity of the Transformer architecture by restricting self-attention computations to non-overlapping local windows of size 7 × 7. While these Transformer models can achieve or surpass CNN accuracy when pretrained on large datasets, they are often parameter-heavy. When scaled down to build lightweight ViT models, their performance significantly lags behind that of CNNs. Mehta et al. [19] introduced a lightweight Transformer structure, termed the MobileViT module, which learns global representations with spatial inductive bias from image features. This module is designed similarly to standard convolutions, replacing the matrix multiplication part of convolution with a deeper Transformer structure, enabling MobileViT to inherit convolution-like spatial bias properties.

**Figure 4:**
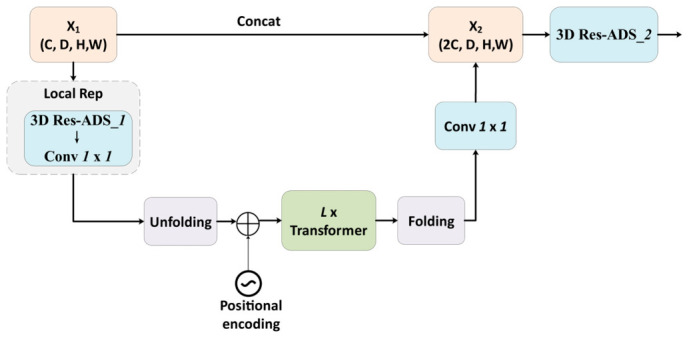
Structure diagram of the LiteViT block. Here, 3DRes-ADS_1 represents a 3DRes-ADS block with a residual connection, while 3DRes-ADS_2 represents a 3DRes-ADS block without a residual connection. In L × Transformer, L denotes the number of stacked Transformer blocks, and L is set to 1 in the implemented LiteViT module used in this study.

Inspired by the excellent design of MobileViT, we improve the MobileViT module by incorporating the 3D Res-ADS axial depthwise separable convolution structure, resulting in the LiteViT module. This modification further reduces the model size while retaining the original advantages and adds learnable positional encodings to the Transformer component to enhance the model’s global modeling capability. The detailed structure of the LiteViT module is shown in Figure 4.

Given a tensor $F\in R^{K\times \frac{H}{16}\times \frac{W}{16}\times \frac{D}{16}}$ (K = 192), a 3D Res-ADS block is used to encode local spatial information, followed by a pointwise convolution that projects the tensor into a high-dimensional space (dimension = 64) through a learned linear combination of the input channels. Next is the unfolding process, which expands the model’s ability to learn global spatial features. The feature map processed by the pointwise convolution is unfolded into N non-overlapping flattened 3D patches, where P=Patch_h×Patch_w×Patch_d (i.e., the volume of the 3D patches, with Patch_h, Patch_w, Patch_d representing the height, width, and depth of the 3D patches, respectively), and N = (H × W × D)/P (i.e., the number of 3D patches obtained after unfolding). Then, one Transformer block, namely (L = 1), is used to encode the relationships among all 3D patches in the LiteViT module. The subsequent folding process can be viewed as the inverse of the unfolding operation. After projecting to a lower-dimensional space via pointwise convolution, the result is concatenated with the original tensor X, followed by a 3D Res-ADS block for feature fusion. The final output tensor has the same shape as the input, with the feature representations *F*.

### 3.5. Decoder Module

To generate segmentation results in the original 3D image space H×W×D, we introduce a 3D CNN decoder for feature upsampling and pixel-level segmentation, with the specific structure shown in Figure 3b. First, an UpSampling module is used for upsampling (in this experiment, trilinear interpolation is applied to reduce the model’s parameter count). To further enhance the U-Net-like model’s ability to recognize multi-scale image features extracted by the Skip Connections module and improve the model’s interpretability, we integrate an Additive Attention Gate [12] into the decoder. The attention coefficients obtained are normalized using a Sigmoid activation function. This is followed by two 3D Res-ADS modules with kernel sizes of 7 × 7 × 7. The specific encoding process is as follows: features from each stage are upsampled, attention is computed after the double convolution operation, a skip connection is applied, and finally, the fused features are passed through a 3D convolution with a kernel size of 1 × 1 × 1 to generate the final segmentation mask.

For TransLiteUNet-S, in order to further reduce the model’s computational cost, this study simplifies its decoder. Specifically, the decoder of TransLiteUNet-S utilizes only a single 3D Res-ADS module, and the attention mechanism in the skip connection is removed while the gating operation is retained. Compared with the base TransLiteUNet, this simplified decoder design further reduces the number of parameters and computational complexity. A complete layer-wise comparison of parameters between TransLiteUNet and TransLiteUNet-S is provided in Table S1 and Table S2, respectively, allowing readers to identify exactly which modules contribute to the parameter reduction. The LiteViT module is consistently implemented with L = 1.

## 4. Experiments

### 4.1. Dataset Description and Model Evaluation Metrics

The two 3D MRI public datasets used in the experiments are provided by the Brain Tumor Segmentation Challenge. The BraTS2020 dataset [39,40,41] consists of 369 training cases, 125 validation cases, and 166 test cases, with the training cases having publicly available label data. The BraTS2019 dataset [39,40,41] comprises 335 training cases and 125 validation cases. Apart from differences in the number of patient image samples, the imaging and labeling information in both datasets remain consistent.

The scan data for each patient includes four different modalities, all with a volume of 240 × 240 × 155. All multimodal scan images are provided in NIFTI file format and consist of the following four distinct modalities: T1-weighted (T1WI) sequence, T1 contrast-enhanced (T1CE) sequence, T2-weighted (T2WI) sequence, and FLAIR sequence. These scans were acquired through different clinical protocols and various scanners from 19 different institutions. The corresponding labels consist of four categories: background region (label 0), necrotic non-enhancing tumor (label 1), peritumoral edema (label 2), and enhancing tumor (label 4). Before release, the data underwent essential preprocessing steps, including co-registration to a common anatomical template, interpolation to a uniform resolution of 1×1×1 mm^3^, and skull stripping.

In addition to the preprocessing steps applied to the dataset itself, we performed additional intensity normalization on the data. Specifically, the non-zero voxel values within each modality were normalized using Z-score normalization. Subsequently, the four image modalities and corresponding label data for each patient were stored in a single HDF5 file. The model’s segmentation accuracy was evaluated using the Dice score for the enhancing tumor region (ET, label 1), tumor core region (TC, labels 1 and 4), and whole tumor region (WT, labels 1, 2, and 4).

### 4.2. Experimental Details

The TransLiteUNet proposed in this paper is implemented in PyTorch 2.0.0 and trained from scratch using an NVIDIA GeForce RTX 4090 GPU (24 GB VRAM). We performed 5-fold cross-validation experiments on the BraTS2020 and BraTS2019 datasets, with data splitting achieved using the KFold function from the Sklearn.model_selection library.

The number of training epochs was set to 1000, and the model optimizer used was SGD with momentum (SGDM), incorporating weight decay. The learning rate scheduling strategy employed was cosine annealing with warmup, with the warmup period set to 20. The loss function used in this study was a weighted combination of cross-entropy loss and Dice loss, which can be formulated as follows:

(3) $$L_{total}=\lambda_{CE}L_{CE}+\lambda_{Dice}L_{Dice}$$

where $L_{CE}$ denotes the cross-entropy loss, which encourages voxel-wise classification accuracy, and $L_{Dice}$ denotes the Dice loss, which is used to alleviate class imbalance and improve the overlap between the predicted segmentation and the ground truth. The two loss terms are defined as follows:

(4) $$L_{CE}=-\frac{1}{N}\sum_{i=1}^{N}\sum_{c=1}^{C}y_{i,c}log(p_{i,c})$$

(5) $$L_{Dice}=1-\frac{1}{C}\sum_{c=1}^{C}\frac{2\sum_{i=1}^{N}p_{i,c}y_{i,c}+\epsilon }{\sum_{i=1}^{N}p_{i,c}+\sum_{i=1}^{N}y_{i,c}+\epsilon }$$

where N represents the number of voxels, C denotes the number of segmentation classes, $p_{i,c}$ is the predicted probability of voxel i belonging to class c, $y_{i,c}$ is the corresponding one-hot encoded ground-truth label, and ϵ is a small constant used to avoid division by zero. In our experiments, the weights of cross-entropy loss and Dice loss were empirically set to $\lambda_{CE}=\lambda_{Dice}=0.5$ respectively.

It is worth noting that annotation noise is a common challenge in medical image segmentation due to inter-annotator variability. Recent studies have explored robust alternatives to standard cross-entropy; for instance, Zhou et al. [42] propose a correntropy-based loss function that effectively suppresses the influence of noisy labels during training. While our current implementation adopts the conventional cross-entropy formulation, integrating such noise-robust loss designs represents a promising direction for further improving model robustness in future work. The training process incorporated the following data augmentation techniques: (1) randomly flipping one axis in the axial, coronal, and sagittal planes during each iteration; (2) randomly cropping all modality data from 240 × 240 × 155 to 128 × 128 × 128 voxels.

### 4.3. Experimental Results

To ensure a fair evaluation of the model’s performance, we conducted 5-fold cross-validation on the training sets of the BraTS2020 public dataset (comprising 369 images with full segmentation labels) and the BraTS2019 dataset (comprising 335 training images). We compared the proposed TransLiteUNet model with previous state-of-the-art 3D image segmentation methods. It should be emphasized that the results of the compared baseline models were not directly copied from their original publications. Instead, each baseline model was retrained by replacing only the network architecture in our unified experimental framework while keeping the same preprocessing pipeline, data split, training strategy, and evaluation protocol. This design ensures that the performance differences mainly reflect the effectiveness of different model architectures under comparable experimental conditions.

The experimental results shown in Table 1 indicate that our TransLiteUNet model achieved Dice scores of 0.769 ± 0.110, 0.850 ± 0.059, 0.911 ± 0.017, and 0.843 ± 0.055 for the ET, TC, WT, and their average, respectively, on the BraTS2020 dataset. The results in Table 2 show that TransLiteUNet achieved Dice scores of 0.766 ± 0.135, 0.839 ± 0.086, 0.908 ± 0.012, and 0.838 ± 0.074 for ET, TC, WT, and their average, respectively, on the BraTS2019 dataset. Compared to prior state-of-the-art methods, our model outperforms in all metrics under the same experimental conditions. Furthermore, it is noteworthy that the number of parameters (Params) and floating-point operations per second (FLOPs) of TransLiteUNet are reduced by at least several tens or even hundreds of times compared to all previous models. This highlights the significant advantages of TransLiteUNet in terms of both model performance and parameter efficiency, primarily attributed to our proposed 3D Res-ADS, LiteViT modules, and the lightweight U-Net network architecture.

To strengthen the statistical reliability of the reported results, we further report the standard deviation of the Dice scores across the five cross-validation folds in Table 1 and Table 2. The standard deviation reflects the performance variation caused by different training and validation splits. It can be observed that TransLiteUNet maintains stable performance across folds and achieves the best or comparable average Dice scores on both BraTS2020 and BraTS2019 datasets.

It should also be noted that the main contribution of this work lies not only in the modest improvement of segmentation accuracy, but also in achieving comparable or superior performance with a substantially smaller model size and lower computational cost. For example, on the BraTS2020 dataset, TransLiteUNet improves the average Dice score from 0.837 ± 0.059, achieved by 3D UX-Net, to 0.843 ± 0.055 while reducing the number of parameters from 53.05 M to 0.43 M and the FLOPs from 1518.81 G to 14.98 G. Therefore, the advantage of TransLiteUNet should be interpreted from both segmentation accuracy and model efficiency.

Although statistical significance tests can be applied to cross-validation results, only five folds are available in this study, and the training subsets of different folds are partially overlapping rather than fully independent samples. Under this setting, conventional significance tests may have limited statistical power and may not provide sufficiently reliable inference. Therefore, instead of over-interpreting small numerical differences, we report the mean and standard deviation across folds and focus on the consistency of performance together with the substantial reduction in model complexity.

Beyond reducing the number of parameters and computational cost, the proposed architectural components are also specifically designed to address several challenges in brain tumor segmentation. First, enhancing tumor regions are often small, irregular, and surrounded by complex tissue structures, making them prone to being missed by models with limited local contextual perception. The proposed 3D Res-ADS module adopts axial depthwise separable convolutions with a large kernel design, which enlarges the effective receptive field while maintaining low computational complexity. This enables the model to capture richer local texture and boundary information around small tumor regions. This design is consistent with the results in Table 1, where TransLiteUNet achieves the highest ET Dice score of 0.769 on the BraTS2020 dataset, indicating its strong sensitivity to small and difficult tumor regions.

Second, edema and whole-tumor regions usually exhibit large spatial extent and irregular shapes, requiring both local boundary perception and global contextual understanding. Pure CNN-based models may suffer from limited long-range dependency modeling, which can lead to fragmented predictions or false-positive regions. In contrast, the LiteViT module combines CNN-based local feature extraction with Transformer-based global dependency modeling. The 3D Res-ADS layer first provides spatially informative tokens, and the Transformer block further models the relationships among 3D patches. In addition, the learnable positional encoding helps preserve spatial location information during global feature interaction. Therefore, TransLiteUNet can better maintain the structural consistency of tumor regions while suppressing isolated false predictions.

**Figure 5:**
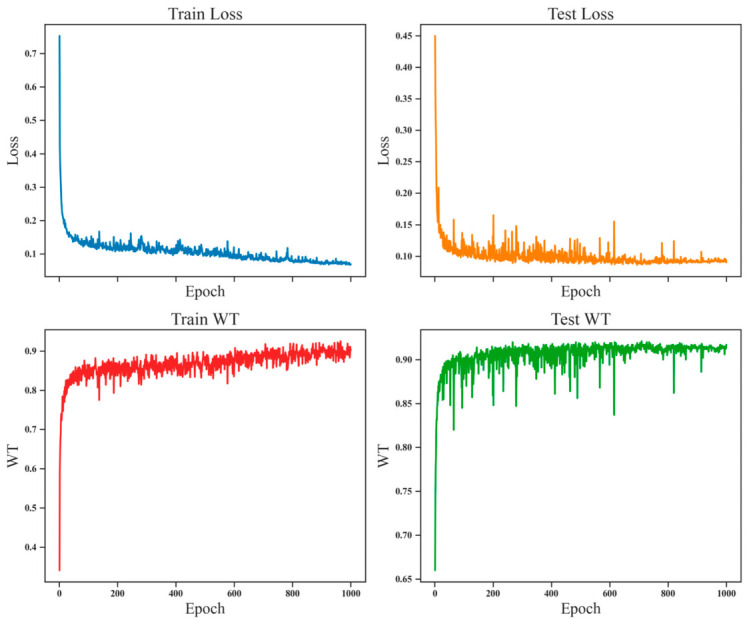
The dynamic changes in different metrics during the training process of the TransLiteUNet model on the BraTS2019 dataset.

**Figure 6:**
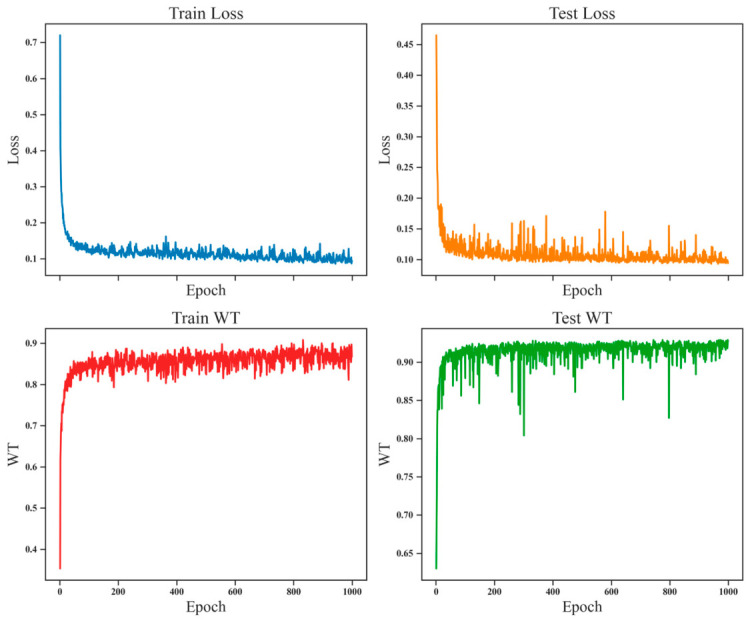
The dynamic changes in different metrics during the training process of the TransLiteUNet model on the BraTS2020 dataset.

To facilitate the observation of metric changes during the training process of TransLiteUNet, this study records the real-time performance of the model during training and presents it as line charts. The real-time changes of the TransLiteUNet model on the BraTS2019 dataset are shown in Figure 5, while the changes on the BraTS2020 dataset are shown in Figure 6. It is evident from the figures that after 500 epochs of training, the model’s loss and WT metrics on the test set gradually converge to their optimal values.

**Figure 7:**
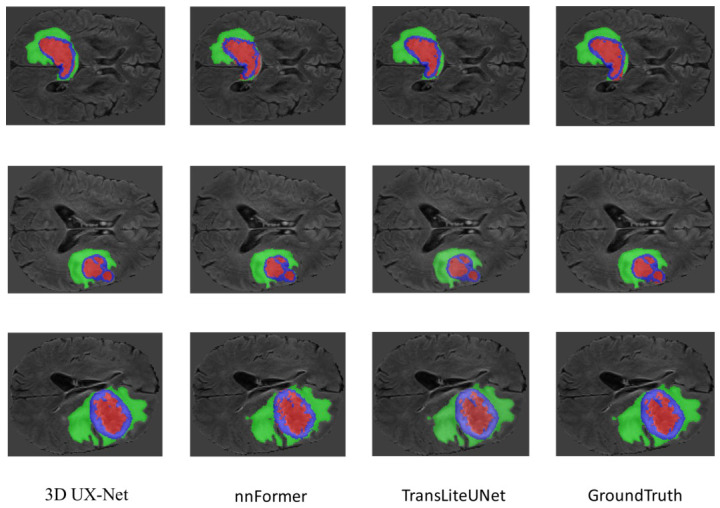
Visual qualitative comparison of partial segmentation results from the BraTS2020 dataset. The blue region represents the enhancing tumor, the red region represents the necrotic and non-enhancing tumor, and the green region represents the peritumoral edema.

**Figure 8:**
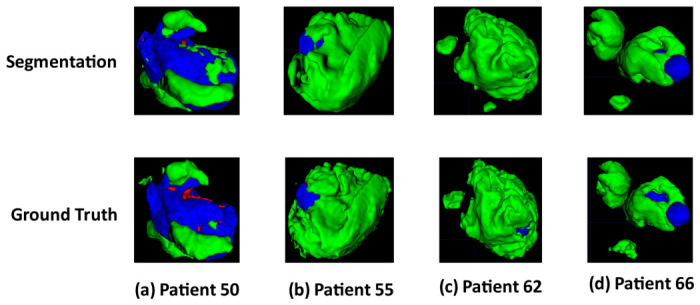
The 3D visualization of partial segmentation results from the BraTS2020 dataset. The first row displays the segmentation results from the TransLiteUNet model, while the second row shows the corresponding segmentation labels. The numbers at the bottom represent the identifiers of different patients. The blue region represents the enhancing tumor, the red region represents the necrotic and non-enhancing tumor, and the green region represents the peritumoral edema.

Furthermore, we performed a visual qualitative analysis of the segmentation results from 3D UX-Net [23], nnFormer [38], and TransLiteUNet, as shown in Figure 7 and Figure 8. These figures display the brain tumor segmentation results from different models. It is evident from the images that although these recently proposed state-of-the-art models yield promising results, TransLiteUNet demonstrates a more precise handling of the segmentation details for brain tumors.

As shown in Figure 7, although 3D UX-Net and nnFormer can generate generally reasonable tumor segmentation results, some local differences can still be observed. For the edema regions, especially in the first and second rows, the compared models tend to show slight over-segmentation or under-segmentation around the green boundary, leading to local contours that deviate from the ground truth. In contrast, TransLiteUNet produces edema boundaries that are more consistent with the ground-truth annotations. For the enhancing tumor region, particularly in the large-lesion case shown in the third row, the blue region predicted by nnFormer appears relatively fragmented or has less regular boundaries, whereas TransLiteUNet generates a more compact and smoother enhancing tumor region that better matches the shape of the ground truth. These differences are also consistent with the quantitative results in Table 1, where TransLiteUNet achieves the highest ET Dice score of 0.769 on the BraTS2020 dataset, indicating that the proposed lightweight design does not weaken its sensitivity to small and difficult tumor regions.

The improved visual consistency of TransLiteUNet can be attributed to the complementary design of the proposed 3D Res-ADS and LiteViT modules. The 3D Res-ADS module effectively captures local spatial details with a large receptive field and low computational cost, which helps preserve fine tumor boundaries. Meanwhile, the LiteViT module enhances global contextual modeling among 3D patches, reducing isolated false predictions and irregular protrusions in the segmentation results. Therefore, TransLiteUNet can generate tumor shapes that are closer to the ground truth while maintaining a lightweight model structure.

## 5. Discussion

### 5.1. Analysis of Model Complexity and Inference Cost

Current mainstream models, such as UNETR [13], CKD-TransBTS [14], and nnFormer [38], have significantly improved the segmentation performance of 3D medical images by leveraging the ViT framework. This research paradigm greatly enhances the global feature modeling ability of the models. However, the drawbacks are also evident. Due to the high computational complexity of pure Transformer architectures, these models experience a substantial increase in parameter scale and complexity, along with higher requirements for the size of training data. This poses a significant challenge in the medical field, where the available data are often limited. When the training data size is insufficient, the performance of these models tends to degrade considerably. Moreover, recently proposed lightweight pure 3D CNN segmentation architectures, such as 3D UX-Net [23], have achieved outstanding results in many medical image segmentation tasks. By employing large 7 × 7 × 7 convolution kernels and depthwise separable convolutions, it enhances the receptive field of the model while reducing the parameter scale. However, its parameter scale and computational complexity still reach 53.05 M and 1518.81 GFLOPs, respectively. The TransLiteUNet model proposed in this paper is an efficient, lightweight 3D image segmentation model with only 0.43 M parameters and 14.98 G FLOPs. This makes it highly competitive compared to almost all previously proposed models in the same category. Detailed parameters for the different models, including their parameters and computational complexity, are provided in Table 1.

Although TransLiteUNet already demonstrates significant advantages, for deployment in mobile device environments, users prefer models that are as lightweight as possible while maintaining superior performance. Therefore, we conducted a statistical analysis of the parameter scale of the different modules in TransLiteUNet. According to the layer-wise breakdown in Table S1, the encoder path (including the initial convolution and down-sampling layers) accounts for only about 0.071 M parameters, while the bottleneck lightweight Transformer module contributes approximately 0.177 M trainable parameters. The decoder path (comprising the attention upsampling layers and the output layer) accounts for around 0.184 M parameters and, together with the bottleneck module, constitutes the main parameter overhead. The relatively large parameter scale of the decoder is primarily due to its use of two 3DRes-ADS modules and the gated attention mechanism in the skip connections. In pursuit of a more lightweight model, we simplified the decoder by retaining only one 3DRes-ADS module and removing the gated attention mechanism in the skip connection while retaining the gating operation. By this streamlined design, TransLiteUNet-S reduces the decoder parameters to about 0.066 M; a detailed per-layer comparison is provided in Table S2. The LiteViT module is consistently implemented with one Transformer block in both TransLiteUNet and TransLiteUNet-S. Therefore, the reduction in parameters and computational complexity of TransLiteUNet-S mainly comes from the simplified decoder design. This simplified model has only 0.31 M parameters and 7.68 G FLOPs, with a marginal decrease in segmentation accuracy compared to the TransLiteUNet model, while still maintaining a performance advantage over other models. Detailed results are presented in Table 1 and Table 2.

In addition to the model’s parameter size and computational complexity, the training time and inference time are also crucial metrics for evaluating model lightweighting. The detailed evaluation metrics are presented in Table 3. The training time of the TransLiteUNet model is 54.00 s per epoch, and the inference time is 0.05 s per sample. For TransLiteUNet-S, the training time is reduced to 34.20 s per epoch, and the inference time is 0.03 s per sample. While maintaining the advantage in segmentation performance, TransLiteUNet shows only a modest improvement in training and inference costs compared to current mainstream models. However, TransLiteUNet-S demonstrates significant optimization over previously proposed segmentation models. This optimization can shorten the development cycle, enhance system response speed, and reduce model deployment costs.

### 5.2. Ablation Study

To further quantify and analyze which modules in TransLiteUNet contribute most significantly to the model’s performance improvement, we conducted a comprehensive ablation study based on the BraTS2020 public dataset using 5-fold cross-validation. The ablation study used the 3D Attention U-Net model design as the baseline and incrementally added the 3D Res-ADS and LiteViT module designs, observing their impact on both model performance and complexity. The results are presented in Table 4. The findings indicate that the 3D Res-ADS module provided the largest improvement over the baseline, with an average Dice score increase of 3.9%, while also achieving a significant reduction in both parameters and FLOPs. The Lite-MobileViT module further enhanced the segmentation accuracy, achieving an additional 1% increase in the average Dice score.

It can be observed that the 3D Res-ADS, a 3D convolution operator developed based on depthwise separable convolutions and residual connections, has significantly enhanced image feature extraction and parameter efficiency. More importantly, this module is particularly suitable for brain tumor segmentation because it helps balance the contradiction between small-lesion sensitivity and computational redundancy. Enhancing tumor regions are usually small, sparse, and morphologically irregular. If only small convolution kernels are used, the model may fail to capture sufficient surrounding context; however, directly using standard large 3D convolution kernels would introduce excessive parameters and computational cost. The proposed 3D Res-ADS module addresses this problem by decomposing the large 3D convolution into axial depthwise separable convolutions, thereby expanding the effective receptive field with limited additional cost. This design enables the model to better perceive local boundary changes and high-gradient tumor edges, which is beneficial for segmenting small and irregular enhancing tumor regions. As shown in Table 4, adding the 3D Res-ADS module improves the ET Dice score from 0.725 to 0.755 and the average Dice score from 0.794 to 0.833, while reducing the parameter size from 6.44 M to 0.25 M.

The LiteViT module further improves the segmentation performance by integrating 3D Res-ADS convolution layers with Transformer layers. Its contribution is not limited to lightweight design; instead, it provides complementary global contextual modeling for 3D brain tumor segmentation. Brain tumors, especially edema and whole-tumor regions, often have irregular spatial distributions and ambiguous boundaries with surrounding normal tissues. Local convolutional features alone may lead to incomplete tumor regions, fragmented predictions, or false positives. In LiteViT, the convolutional component first extracts local spatial features and introduces strong inductive bias, while the Transformer component models long-range dependencies among 3D patches. This hybrid design allows the model to simultaneously preserve detailed tumor boundaries and understand the global structural relationship of tumor regions. The learnable positional encoding further enhances spatial awareness during global feature interaction. As shown in Table 4, after adding LiteViT, the ET, TC, WT, and average Dice scores are further improved to 0.769, 0.850, 0.911, and 0.843, respectively. These results indicate that the proposed architectural changes are beneficial not only for reducing model complexity but also for improving the segmentation of small lesions and maintaining the structural completeness of tumor regions.

### 5.3. Potential Extension to Brain Tumor Classification

Although this study mainly focuses on brain tumor segmentation, we agree that tumor classification is also a clinically important task. For example, distinguishing different glioma subtypes or grades, such as low-grade glioma and glioblastoma, can provide valuable information for clinical diagnosis, treatment planning, and prognosis assessment. Since the present work is based on the BraTS segmentation setting, our experiments primarily evaluate voxel-level tumor delineation. Nevertheless, the proposed TransLiteUNet architecture has the potential to be extended to brain tumor classification tasks.

Specifically, the encoder and LiteViT module of TransLiteUNet can be regarded as a lightweight 3D feature extraction backbone. The 3D Res-ADS module can capture local texture, boundary, and morphological features of tumor regions, while the LiteViT module can further model global contextual relationships among 3D patches. These properties are also beneficial for tumor classification because classification requires not only local lesion characteristics but also global structural information from multi-modal MRI scans. To adapt TransLiteUNet for classification, the decoder can be replaced with a lightweight classification head composed of convolutional layers, global pooling, and fully connected layers. In this way, the model can output image-level tumor categories instead of voxel-level segmentation masks.

In future work, we will further investigate the classification capability of TransLiteUNet on datasets with image-level pathological, molecular, or tumor-grade annotations. We also plan to explore joint segmentation–classification learning, where the segmentation branch provides tumor localization and structural information, while the classification branch predicts tumor subtype or grade. Such a multi-task framework may further improve the clinical applicability of the proposed model.

## 6. Conclusions

This paper proposes a novel 3D lightweight segmentation framework, TransLiteUNet. The introduced 3D Res-ADS module is designed to reduce the model’s complexity and enhance its parameter efficiency, while the LiteViT module adds the capability for global information modeling. TransLiteUNet does not require any pretraining, and while achieving a slight improvement in segmentation accuracy, its parameter size and FLOPs are only 0.43 M and 14.98 G, respectively, representing a reduction of several tens or even hundreds of times compared to previous models. Furthermore, we further simplified the design of TransLiteUNet and proposed TransLiteUNet-S, which has only 0.31 M parameters and 7.68 G FLOPs, providing significant advantages in terms of training and inference costs. To the best of our knowledge, this is the model with the lowest parameter count and model complexity among all existing 3D image segmentation models. TransLiteUNet has achieved significant success in terms of model lightweighting. In future work, we plan to expand the use cases of TransLiteUNet to accommodate multi-organ segmentation tasks, aiming to establish it as a universal model benchmark in the field of 3D image segmentation. In addition, we will explore the extension of TransLiteUNet to brain tumor classification tasks by using the encoder and LiteViT module as a lightweight 3D feature extraction backbone and replacing the segmentation decoder with a classification head. This extension may enable the model to support clinically relevant tasks such as tumor subtype or grade prediction. Additionally, we will continue to explore improvements in the global feature modeling capabilities of TransLiteUNet and the lightweighting of CNN modules, striving to achieve and exceed the performance levels of other mainstream models at a lower computational cost.

## Figures and Tables

**Table 1:** Comparison of the average results on the validation set of the BraTS2020 dataset using 5-fold cross-validation for different models. The Dice scores are reported as mean ± standard deviation across the five folds. Arrows indicate the optimal direction (↑: higher is better, ↓: lower is better).

Method	Params ↓	FLOPs ↓	Dice Score ↑			
			ET	TC	WT	AVG
3D-Unet	10.05 M	382.11 G	0.742 ± 0.095	0.813 ± 0.068	0.889 ± 0.025	0.815 ± 0.061
3D Attention U-Net	6.44 M	301.71 G	0.725 ± 0.106	0.772 ± 0.104	0.884 ± 0.028	0.794 ± 0.069
nnFormer	149.10 M	521.05 G	0.721 ± 0.095	0.795 ± 0.084	0.894 ± 0.013	0.803 ± 0.061
CKD-TransBTS	81.60 M	462.60 G	0.748 ± 0.119	0.827 ± 0.071	0.903 ± 0.011	0.826 ± 0.064
SwinUNETR	61.99 M	794.02 G	0.742 ± 0.120	0.814 ± 0.077	0.902 ± 0.016	0.819 ± 0.068
3D UX-Net	53.05 M	1518.81 G	0.755 ± 0.120	0.846 ± 0.064	0.910 ± 0.018	0.837 ± 0.059
UNETR	102.06 M	203.36 G	0.732 ± 0.127	0.804 ± 0.077	0.900 ± 0.014	0.812 ± 0.066
TransLiteUNet	0.43 M	14.98 G	0.769 ± 0.110	0.850 ± 0.059	0.911 ± 0.017	0.843 ± 0.055
TransLiteUNet-S	0.31 M	7.68 G	0.762 ± 0.077	0.846 ± 0.055	0.912 ± 0.015	0.840 ± 0.044

**Table 2:** Comparison of the average results on the validation set of the BraTS2019 dataset using 5-fold cross-validation for different models. The Dice scores are reported as mean ± standard deviation across the five folds. Arrows indicate the optimal direction (↑: higher is better, ↓: lower is better).

Method	Params ↓	FLOPs ↓	Dice Score ↑			
			ET	TC	WT	AVG
nnFormer	149.10 M	521.05 G	0.716 ± 0.151	0.800 ± 0.116	0.885 ± 0.017	0.800 ± 0.094
3D UX-Net	53.05 M	1518.81 G	0.749 ± 0.161	0.840 ± 0.083	0.904 ± 0.013	0.831 ± 0.082
UNETR	102.06 M	203.36 G	0.727 ± 0.150	0.809 ± 0.082	0.895 ± 0.015	0.810 ± 0.078
TransLiteUNet	0.43 M	14.98 G	0.766 ± 0.135	0.839 ± 0.086	0.908 ± 0.012	0.838 ± 0.074
TransLiteUNet-S	0.31 M	7.68 G	0.773 ± 0.137	0.827 ± 0.085	0.906 ± 0.012	0.835 ± 0.073

**Table 3:** Comparison of training time (in seconds per epoch) and inference time (in seconds per sample) of different models on the BraTS2020 dataset. Arrows indicate the optimal direction (↓: lower is better).

Evaluation Metrics	nnFormer	UNETR	3D UX-Net	TransLiteUNet	TransLiteUNet-S
Training Time ↓	64.20	55.80	236.40	54.00	34.20
Inference Time ↓	0.05	0.04	0.20	0.05	0.03

**Table 4:** Ablation study results, showing the validation set results of the 5-fold cross-validation on the BraTS2020 dataset. The baseline model is a 3D Attention U-Net. The Dice scores are reported as mean ± standard deviation across the five folds. Arrows indicate the optimal direction (↑: higher is better, ↓: lower is better).

Method	Dice Score ↑				Params ↓	FLOPs ↓
	ET	TC	WT	AVG		
Baseline	0.725 ± 0.106	0.772 ± 0.104	0.884 ± 0.028	0.794 ± 0.069	6.44 M	301.71 G
Baseline + 3D Res-ADS	0.755 ± 0.122	0.836 ± 0.067	0.908 ± 0.015	0.833 ± 0.063	0.25 M	14.89 G
Baseline + 3D Res-ADS + LiteViT	0.769 ± 0.110	0.850 ± 0.059	0.911 ± 0.017	0.843 ± 0.055	0.43 M	14.98 G

## Data Availability

The datasets generated and analyzed during the current study are available from the corresponding author upon reasonable request.

## Supplementary Materials

The following supporting information can be downloaded at: https://www.mdpi.com/article/10.3390/jimaging12070290/s1, Table S1: Parameters of each layer in TransLiteUNet; Table S2: Parameters of each layer in TransLiteUNet-S.

## Abbreviations

The following abbreviations are used in this manuscript:

CNNs	Convolutional Neural Networks
FCNs	Fully Convolutional Networks
ViT	Vision Transformer
T1WI	T1-Weighted Sequence
T1CE	T1 Contrast-Enhanced Sequence
T2WI	T2-Weighted Sequence
SGDM	SGD with Momentum
