# Supplementary material for: TransLiteUNet: A Lightweight CNN–Transformer Hybrid for Efficient 3D Brain Tumor Segmentation with Sub-0.5 M Parameters
Source: J Imaging. 2026 Jun 30;12(7):290. doi: 10.3390/jimaging12070290 (PMC13412664; doi:10.3390/jimaging12070290)
Supplement: Supplementary file 1 [file jimaging-12-00290-s001.zip › jimaging-4349371-supplementary.pdf]

Table S1. Parameters of each layer in TransLiteUNet

Layer	Output Shape	Params	Trainable params
InConv-1	[1, 24, 128, 128, 128]	236	236
Down-2	[1, 48, 64, 64, 64]	1,800	1,800
Down-3	[1, 96, 32, 32, 32]	5,904	5,904
Down-4	[1, 192, 16, 16, 16]	21,024	21,024
Down-5	[1, 192, 8, 8, 8]	41,664	41,664
MobileViTBlock-6	[1, 192, 8, 8, 8]	177,264	177,264
AttentionUpBlock-7	[1, 96, 16, 16, 16]	132,194	132,194
AttentionUpBlock-8	[1, 48, 32, 32, 32]	36,146	36,146
AttentionUpBlock-9	[1, 24, 64, 64, 64]	10,586	10,586
AttentionUpBlock-10	[1, 24, 128, 128, 128]	4,754	4,754
OutConv-11	[1, 4, 128, 128, 128]	100	100
**Total**		**431,672 (0.43M)**	**431,672 (0.43M)**

Table S2. Parameters of each layer in TransLiteUNet-S

Layer	Output Shape	Params	Trainable params
InConv-1	[1, 24, 128, 128, 128]	236	236
Down-2	[1, 48, 64, 64, 64]	1,800	1,800
Down-3	[1, 96, 32, 32, 32]	5,904	5,904
Down-4	[1, 192, 16, 16, 16]	21,024	21,024
Down-5	[1, 192, 8, 8, 8]	41,664	41,664
MobileViTBlock-6	[1, 192, 8, 8, 8]	177,264	177,264
AttentionUpBlock-7	[1, 96, 16, 16, 16]	45,888	45,888
AttentionUpBlock-8	[1, 48, 32, 32, 32]	13,728	13,728
AttentionUpBlock-9	[1, 24, 64, 64, 64]	4,560	4,560
AttentionUpBlock-10	[1, 24, 128, 128, 128]	2,304	2,304
OutConv-11	[1, 4, 128, 128, 128]	100	100
**Total**		**314,472 (0.31M)**	**314,472 (0.31M)**
